# Das Anti-IgLON5-Syndrom in der klinischen Neurologie – zwei Fallberichte

**DOI:** 10.1007/s00115-022-01344-9

**Published:** 2022-06-15

**Authors:** Johanna Niederschweiberer, Nicolas U. Schumacher, Daniela Kumpfmüller, Charlotte Lingg, Simone Graf, Benno Ikenberg, Mark Mühlau, Paul Lingor, Bernhard Hemmer, Benjamin Knier

**Affiliations:** 1 Klinik und Poliklinik für Neurologie, Klinikum rechts der Isar, Technische Universität München, Ismaninger Str. 22, 81675 München, Deutschland; 2 Klinik für Anästhesiologie und Intensivmedizin, Klinikum rechts der Isar, Technische Universität München, München, Deutschland; 3 Klinik und Poliklinik für Hals-Nasen-Ohrenheilkunde, Klinikum rechts der Isar, Technische Universität München, München, Deutschland; 4 Munich Cluster for Systems Neurology (SyNergy), München, Deutschland

## Hintergrund

Störungen der Vigilanz, des Schlafes sowie verschiedener Hirnstammfunktionen finden sich bei einer Vielzahl neurologischer Erkrankungen. Neben den häufigen Differenzialdiagnosen der einzelnen Symptome sollten dabei, insbesondere beim gemeinsamen Auftreten im Rahmen eines Syndroms, auch seltene Ursachen erwogen werden. Erst vor wenigen Jahren konnten Autoantikörper gegen das neuronale Zelladhäsionsmolekül IgLON5 bei Patienten mit Schlafstörungen, Bewegungsstörungen und Hirnstammsymptomen in Serum und Liquor nachgewiesen werden [10]. Die mit dem Nachweis von Anti-IgLON5-Antikörpern beschriebenen klinischen Defizite werden seither als Anti-IgLON5-Syndrom oder Anti-IgLON5-Erkrankung zusammengefasst und als weitere Autoimmunenzephalitis eingeordnet [10].

Die zwei folgenden Fallbeschreibungen sollen nicht nur das heterogene klinische Spektrum des Anti-IgLON5-Syndroms illustrieren, sondern auch aussichtsreiche Therapiemöglichkeiten aufzeigen.

## Kasuistik 1

Eine 79-jährige Patientin stellte sich notfallmäßig mit akuter Dyspnoe in unserer Klinik vor. An relevanten Vorerkrankungen bestanden ein obstruktives Schlafapnoesyndrom (OSAS) und ein arterieller Hypertonus. Bei rasch progredientem inspiratorischem Stridor und bei akutem Laryngospasmus erfolgte die notfallmäßige Intubation mit Aufnahme auf die Intensivstation. Nach der Gabe von Prednisolon und blandem Lokalbefund konnte die Patientin am Folgetag extubiert werden. In der Folge kam es zu wiederholten Laryngospasmen, sodass die Reintubation mit Tracheotomie erfolgte. In einer HNO-ärztlichen Abklärung ergaben sich keine erklärenden Ursachen für die rezidivierenden Laryngospasmen, sodass die Patientin konsiliarisch neurologisch vorgestellt wurde. Fremdanamnestisch wurden eine progrediente Muskelschwäche mit Dysarthrie während der vergangenen Wochen sowie rezidivierende optische Halluzinationen mit Schlafstörungen (sehr lebhafte Träume mit Umherschlagen im Schlaf, a.e. einer REM-Schlaf-Verhaltensstörungen entsprechend) seit Monaten berichtet. Bei der Verlegung auf unsere neurologische Überwachungsstation war die Patientin wach, über ein Tracheostoma spontanatmend und befolgte Aufforderungen. Es fanden sich Atrophien und Faszikulationen an den oberen Extremitäten, Faszikulationen im Gesichtsbereich, Zungenfibrillationen (siehe Video Zusatzmaterial online) sowie in der flexiblen endoskopischen Evaluation des Schluckaktes (FEES) Faszikulationen der Rachenhinterwand, eine oropharyngeale Dysphagie sowie eine Stimmlippenparese. Die Muskeleigenreflexe waren seitengleich und mittellebhaft auslösbar. Ein cMRT zeigte lediglich diskrete mikroangiopathische Marklagerveränderungen. Bei blandem Liquorbefund (1 Zelle/µl, keine Schrankenstörung oder intrathekale Immunglobulinsynthese), regelrechtem EEG und normwertiger Serienstimulation des N. facialis ergaben sich keine Hinweise für eine Hirnstammpathologie, eine Myasthenie oder epileptische Anfälle.

Differenzialdiagnostisch wurde eine Motoneuronerkrankung erwogen, hierzu passend fanden sich pathologische Spontanaktivität im EMG der oberen und unteren Extremitäten und erhöhte Neurofilamentleichtketten im Serum (Nf‑L 204 pg/ml), sodass eine Therapie mit Riluzol begonnen wurde.

Im kurzfristigen Verlauf war bei Hyperkapnie und hiervon unabhängiger fluktuierender Vigilanzminderung eine erneute Verlegung auf unsere Intensivstation nötig. In der erweiterten Differenzialdiagnostik wurden hochtitrige IgLON5-Antikörper im Serum nachgewiesen (1:1000). In der HLA-Genotypisierung zeigten sich die bei der Anti-IgLON5-Erkrankung gehäuft auftretenden Allele HLA-DRB1*1001 und HLA-DQB1*0501.

Unter der Annahme einer Autoimmunenzephalitis mit Anti-IgLON5-Antikörpern erfolgten aufgrund der ausgeprägten Symptomatik und der akuten klinischen Dynamik insgesamt sieben Plasmapheresen (alle zwei Tage), unter denen sich die Symptomatik deutlich besserte. Die Therapie mit Riluzol wurde beendet. Die Kommunikation wurde mittels Sprechkanüle zunehmend verständlich möglich, im Verlauf konnte die Patienten in die Frührehabilitation verlegt werden. Acht Wochen später wurde eine immunmodulatorische Therapie mit Rituximab begonnen. Zu diesem Zeitpunkt präsentierte sich die Patientin mit gebesserter Zungenfibrillation, Dysarthrie und Dysphagie, wobei bei vorbestehendem OSAS und wiederholten Hyperkapniephasen eine nächtliche CPAP-Beatmung initiiert wurde. Hierunter kam zu einer Stabilisierung des OSAS. Zu erneuten Hyperkapniephasen oder REM-Schlaf-Verhaltensstörungen kam es nicht.

## Kasuistik 2

Ein 62-jähriger Patient wurde wegen seit vier Monaten progredienten, wenngleich fluktuierenden Doppelbildern stationär aufgenommen. Klinisch zeigten sich wechselnde Augenmuskelparesen, die mit einer Beteiligung des III., IV. und VI. Hirnnerven vereinbar waren. Weitere fokal-neurologische Defizite waren nicht zu erheben. Zudem klagte der Patient neben Nachtschweiß über eine zunehmende Fatigue-Symptomatik mit Abnahme physischer und kognitiver Belastbarkeit. Bei Angaben einer Schlafstörung konnte im Schlaflabor ein OSAS objektiviert werden. Im cMRT zeigte sich eine Kontrastmittelaufnahme beider Nn. oculomotorii, im Liquor waren eine lymphomonozytäre Pleozytose (13 Zellen/µl) mit erhöhtem B‑Zell- und Plasmablastenanteil (CD19^+^-Zellen 4,8 %, CD19^+^CD138^+^-Zellen 1,3 %) sowie eine Schrankenstörung (Liquor-Serum-Albumin-Quotient 17,9 × 10^−3^) auffällig. Die weitere Diagnostik (Labor, CT-Thorax, Elektrophysiologie) erbrachte keinen wegweisenden Befund. Jedoch fanden sich in der Autoimmunserologie IgLON5-Antikörper (Liquor 1:320, Serum 1:3200).

Bei milder klinischer Symptomatik und langsamer klinischer Dynamik begannen wir eine Therapie mit Prednisolon (oral 1 mg/kgKG) für insgesamt 18 Tage. Bei ambulanten Vorstellungen waren die Symptome gut zurückgebildet; nach 5 Monaten bestanden lediglich noch passagere, morgendliche Doppelbilder. Nach einem Jahr wurde mit Ausnahme eines persistierenden OSAS eine vollständige Beschwerdefreiheit berichtet. Eine nächtliche CPAP-Versorgung wurde vom Patienten abgelehnt. Bei weitgehender Beschwerderemission sahen wir von einer weiteren immunmodulatorischen Langzeittherapie ab.

## Diskussion

Die beiden beschriebenen Fälle zeigen das heterogene klinische Bild der Anti-IgLON5-Erkrankung. Wie im aktuellen Beitrag ersichtlich und in der Literatur beschrieben, umfasst sie neben OSAS häufig Schlafstörungen mit REM-Schlaf-Parasomnien und Bewegungsstörungen [3, 5, 7, 8]. Andere fokal neurologische Defizite wie Laryngospasmen, Bulbärsymptome, autonome, kognitive und psychiatrische Störungen sind ebenfalls häufig [1, 4, 10]. Optische Halluzinationen – wie in Kasuistik 1 aufgetreten – sind bisher nur in Einzelfällen beschrieben [2].

Dem aktuellen Verständnis nach handelt es sich bei der Anti-IgLON5-Erkrankung am ehesten um eine Autoimmunenzephalitis mit neurodegenerativer Komponente. IgLON5 ist ein ubiquitär exprimiertes Zelladhäsionsprotein, dessen genaue neuronale Funktion nicht bekannt ist [11]. In experimentellen Studien konnte ein direkter pathophysiologischer Effekt von Anti-IgLON5-Antikörper auf die Integrität des neuronalen Zytoskeletts gezeigt werden, was die Hypothese einer Enzephalitis unterstreicht [9]. Hierfür spricht auch, wie bei unserer ersten Patientin, die Assoziation mit den HLA-Allelen HLA-DRB1*1001 und HLA-DQB1*0501 [4, 10], was eine genetische Suszeptibilität für autoinflammatorische Vorgänge, wie sie bei einer Vielzahl von Autoimmunerkrankungen beschrieben wurden, nahelegt. Zudem belegt der histologische Nachweis des neuronalen Zelluntergangs mit phosphorylierten Tau-Ablagerungen im Bereich des Tegmentums und Hypothalamus eine, möglicherweise sekundäre, Neurodegeneration [6, 10].

In der Diagnostik der Anti-IgLON5-Erkrankung finden sich darüber hinaus meist unspezifische Befunde. Das cMRT ist meistens unauffällig. Bei bis zu 50 % der Patienten finden sich im Liquor eine Pleozytose und Proteinerhöhung [4]. Antikörper gegen IgLON5 waren bei allen in der Literatur beschriebenen Patienten im Serum und zum Großteil auch im Liquor nachweisbar [1].

Bei den hier beschriebenen Patienten kam es durch die eingeleitete Immuntherapie zu einer deutlichen Symptombesserung. Insgesamt sprechen 30–50 % der Patienten mit Anti-IgLON5-Erkrankung auf Immuntherapien (Steroide, Immunglobuline oder Plasmapheresen) an, wobei eine frühe Immuntherapie mit einem besseren Outcome assoziiert ist [7]. Die Gabe von Second-line-Immuntherapeutika oder eine Kombinationstherapie scheinen das Ansprechen zu erhöhen [1, 7]. Die Wahl der Immuntherapie sollte aus Sicht der Autoren unter Berücksichtigung von Komorbiditäten, Schwere des klinischen Befundes und klinischer Dynamik erfolgen. So entschieden wir uns bei Patientin 1, die bei akuter Dynamik einer wiederholten intensivmedizinischen Therapie bedurfte, für eine Plasmaseparation mit einer Rituximab-Erhaltungstherapie, während beim Patienten 2 bei milder Klinik und langsamer klinischer Dynamik über Monate eine orale Steroidtherapie zur Anwendung kam.

## Fazit für die Praxis

- Die klinischen Manifestationen der Anti-IgLON5-Erkrankung sind heterogen und umfassen Hirnstammsymptome bis hin zur Ateminsuffizienz. Häufig treten Schlafstörungen (Non-REM-Schlaf‑, REM-Schlaf-Verhaltensstörungen, OSAS), auf.
- Entscheidend ist der serologische Nachweis von Anti-IgLON5-Antikörpern. Bei klinischem Verdacht, d. h. vor allem bei bulbären Zeichen, Schlaf(verhaltens)störungen oder Bewegungsstörungen im höheren Lebensalter, sollte diese Testung gerade bei fehlenden Auffälligkeiten in cMRT und Liquor erfolgen.
- Bis zu 50 % der Betroffenen sprechen auf Immuntherapien (Steroide, Immunglobuline, Plasmapherese/Immunadsorption) an.

## Supplementary Information




